# Recovery of Crystallographic Texture in Remineralized Dental Enamel

**DOI:** 10.1371/journal.pone.0108879

**Published:** 2014-10-31

**Authors:** Samera Siddiqui, Paul Anderson, Maisoon Al-Jawad

**Affiliations:** Institute of Dentistry, Barts and The London School of Medicine and Dentistry, Queen Mary University of London, London, United Kingdom; Griffith University, Australia

## Abstract

Dental caries is the most prevalent disease encountered by people of all ages around the world. Chemical changes occurring in the oral environment during the caries process alter the crystallography and microstructure of dental enamel resulting in loss of mechanical function. Little is known about the crystallographic effects of demineralization and remineralization. The motivation for this study was to develop understanding of the caries process at the crystallographic level in order to contribute towards a long term solution. In this study synchrotron X-ray diffraction combined with scanning electron microscopy and scanning microradiography have been used to correlate enamel crystallography, microstructure and mineral concentration respectively in enamel affected by natural caries and following artificial demineralization and remineralization regimes. In particular, the extent of destruction and re-formation of this complex structure has been measured. 2D diffraction patterns collected at the European Synchrotron Radiation Facility were used to quantify changes in the preferred orientation (crystallographic texture) and position of the (002) Bragg reflection within selected regions of interest in each tooth slice, and then correlated with the microstructure and local mineral mass. The results revealed that caries and artificial demineralization cause a large reduction in crystallographic texture which is coupled with the loss of mineral mass. Remineralization restores the texture to the original level seen in healthy enamel and restores mineral density. The results also showed that remineralization promotes ordered formation of new crystallites and growth of pre-existing crystallites which match the preferred orientation of healthy enamel. Combining microstructural and crystallographic characterization aids the understanding of caries and erosion processes and assists in the progress towards developing therapeutic treatments to allow affected enamel to regain structural integrity.

## Introduction

Dental enamel is a unique, highly mineralized biological hard tissue comprised of 96 wt% mineral present in the form of substituted carbonated hydroxyapatite (HA) [Ca_10-x_(PO_4_)_6-x_(CO_3_)_x_(OH)_2_] [1], with traces of organic material and fluid. On the microscopic length-scale, bundles (prisms) of oriented apatite crystallites align relative to each other at acute angles to the enamel dentine junction (EDJ), with less ordered interprismatic enamel in-between. Within prisms, these crystallites are more aligned to each other in the core of the bundle, and less ordered close to the boundaries [2]. At the nanometre length-scale, the apatite crystallites themselves grow into a needle-like shape which displays strong preferred orientation along the *c* axis (growth direction). This intricate multiple length-scale organized structure allows enamel to fulfil its functionality, resulting in a material with oriented strength and hardness for the compressive and shear forces it undergoes during mastication [3].

Early-stage tooth decay (dental caries) is an important pathological disease of enamel which is a worldwide problem affecting 60–90% of the population [4]. It results in the loss of dental tissue, causing the strength and structural integrity to become compromised. In the oral environment, demineralization and remineralization occur constantly either simultaneously or alternately [5]. When the environment becomes acidic (i.e. falls below the critical pH), demineralization becomes the dominant process causing enamel mineral to be lost through dissolution of the apatite crystallites. Generally, in a natural incipient caries lesion, a subsurface lesion is formed 30–135 µm deep [6] into the bulk of the enamel, enclosed by an intact surface zone. The body of the lesion suffers a 20–50% mineral loss [7]. Acidic buffers (for example, acetic acid or lactic acid [8]) can be used to create *in vitro* artificial caries-like lesions but may not necessarily produce a subsurface lesion (as it is isolated from any sort of remineralizing conditions), in which the surface of the enamel is etched away, displaying a steady gradient of dissolution from the enamel surface towards the bulk of the enamel [9].

Enamel is unable to undergo cellular regeneration after suffering damage and thus relies solely upon physico-chemical repair mechanisms to re-establish its original full mineral concentration [10]. Remineralization of early-stage carious lesions can ultimately reverse this damage caused during demineralization; either by re-incorporating lost Ca^2+^ and PO_4_^3−^ back into the crystallite structure sourced from saliva, dental care products, or by formation of new crystallites [11]. Voids and surface roughness on demineralized surfaces and partially dissolved crystallites serve as nucleating sites for *de novo* formation of crystallites or regrowth of existing crystallite structures [12].

Most studies have used conventional microscopy techniques to focus on the qualitative changes in the microstructure of enamel affected by demineralization and remineralization [12]–[14]. Early studies have identified the characteristic changes in structure and mineral concentration in carious lesions. However, quantifying the crystallographic parameters of the apatite crystallites can give insights into the effect this process has on the nano and sub-nano metre length-scales (such as preferred orientation and lattice parameters respectively). Moreover, in a hierarchical structure, comparing physical characteristics at different length-scales with spatial mineral density variations can give a deeper insight into the intricate processes occurring during de- and remineralization of the enamel hard tissue.

The macroscopic mechanical properties of crystalline materials depend on the microscopic and crystallographic arrangement [15]. Texture (or preferred orientation) is a crystallographic term used to describe the arrangement and distribution of orientation directions in a crystalline material. Highly textured materials exhibit organized arrangements of crystallites, whereas materials in which the crystallites are completely randomly oriented have no texture.

Due to the mineral growth process during amelogenesis, enamel is a highly textured material with the strongest preferred orientation in the (002) direction (which is perpendicular to the *c* axis of a hexagonal enamel crystallite). Understanding the texture properties in dental enamel can prove to be useful not only in understanding the pathway of destruction but more importantly the mechanism of reconstruction. Previous texture studies conducted on human teeth have shown enamel to be an anisotropic material, with greatest texture at the surfaces [16] and at the cusps with preferred orientation reducing towards the EDJ [17].

Two-dimensional synchrotron X-ray diffraction (SXRD) is a proven technique to quantify the direction and magnitude of the crystallographic preferred orientation in dental enamel and has been used previously to study the spatial distribution of preferred orientation, lattice parameters, and crystallite size in healthy enamel [18], in enamel affected by genetic disease [19], and in developing human enamel [20].

Small angle X-ray scattering (SAXS) and wide angle X-ray diffraction (WAXRD) have become established techniques for studying the nanostructure of enamel affected by dental caries [21]–[23]. The successful quantification of density and arrangement of apatite crystallites after demineralization [24] and remineralization [25] through the presence and absence of voids respectively, has provided a broader understanding of the caries process at a structural level. Scanning microradiography (SMR) is a directly quantitative X-ray absorption method for measuring the mineral concentration in planoparallel hard tissue slices and has previously been used to measure the changes in projected mineral mass during the caries process as a function of time and position [26]–[30]. The combination of these powerful techniques can provide a greater depth of understanding of the structural changes occurring during de- and remineralization.

To date, little attention has been given to recovering lost preferred orientation of the apatite crystallites, which is crucial for restoring normal and lasting function of the enamel tissue. We therefore present here the application of novel suite of X-ray techniques to understand the crystallographic features of de- and remineralized enamel by comparison with healthy enamel, thus exploring the demineralization pathway, the mechanism of remineralization and characteristics of the remineralized hard tissue.

## Materials and Methods

### 2.1 Sample preparation

Whole tooth specimens were collected with written consent from patients undergoing routine orthodontic extraction at Barts and The London Dental Hospital (approved by the Queen Mary University of London Research Ethics Committee, UK). The extracted teeth were stored in 70% ethanol, and then washed with distilled water before use.

**Figure 1:**
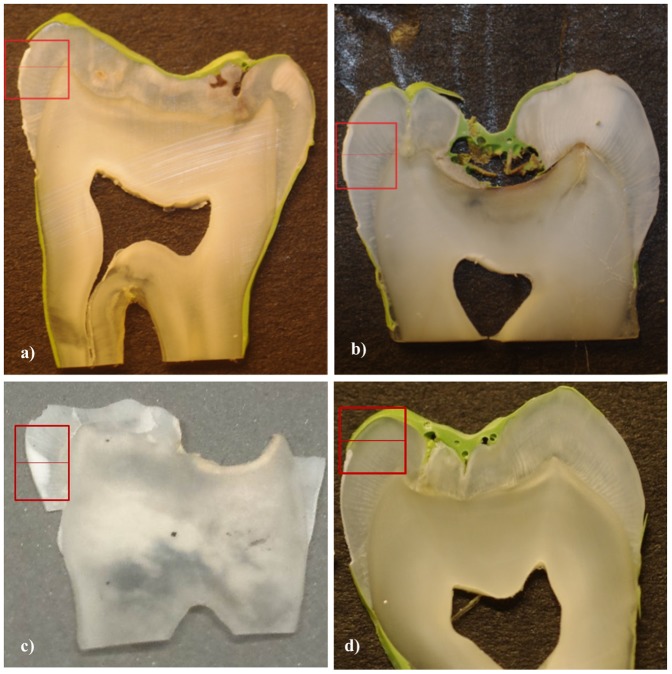
Digital photographs of enamel samples a) artificially demineralized, b) artificially remineralized and c) naturally carious and d) healthy enamel, outlining regions of interest (box) selected for SMR measurement. The line within the box represents the track measured by SXRD.

Four type-matched permanent human mandibular first molar tooth samples were included in this study (Fig. 1); a) enamel with an artificially demineralized lesion; b) artificially demineralized then remineralized enamel; c) naturally carious enamel (identified by the presence of a white spot lesion); and d) a healthy enamel region. For consistency and direct comparability, the same tooth type was used, and a region of interest within each tooth was chosen to be spatially equivalent (see boxes in Fig. 1). The healthy region was selected by ensuring it was not in close proximity to any lesions.

The details of each specimen treatment including the demineralizing and the remineralizing solutions are given in Table 1. Artificial lesions were created by coating a whole healthy tooth crown in acid resistant varnish leaving a 2.0 mm×2.0 mm window of enamel exposed. The whole tooth samples were then sectioned into 500 µm thick longitudinal slices perpendicular to the exposed area using a wet precision annular saw.

**Table 1:** Treatment regimen for tooth samples.

Sample	Treatment	Ingredients	Time
**Artificially Demineralized**	Demineralization	Exposed to 100 ml of 1 vol% acetic acid, buffered to pH 4 using NaOH at room temperature	7 days
**Artificially Remineralized**	Demineralization then Remineralized	Exposed to 100 ml of demineralizing solution, then immersed in 100 ml of remineralizing solution (2 mmol/l CaCl_2_, 1.2 mmol/l KH_2_PO_4_, 150 mmol/l NaCl, 0.05 mmol/l NaF)	7 days
			7 days
**Natural caries**	No treatment	No treatment	NA
**Healthy**	No treatment	No treatment	NA

### 2.2 Synchrotron X-ray diffraction – Crystallography and Nanostructure

#### 2.2.1 Experimental method

2D X-ray diffraction patterns were collected on the BM28 (XMaS) beamline at the European Synchrotron Radiation Facility (ESRF), Grenoble, France. Due to the high user demand of synchrotron facilities, which limits the number of samples and experimental repeats, four teeth were subjected to X-ray diffraction experiments. An X-ray wavelength of 0.82 Å (equivalent to energy 15.1 keV) was used with a sample to detector distance of ∼180 mm allowing a 2θ range of 5–25°. A microfocussed 20.0 µm×20.0 µm beam spot (focussed using vacuum tube slits) was directed on to the mounted sample. A MAR CCD detector (2048×2048 pixels) was placed directly behind the sample and perpendicular to the beam in order to collect images in transmission geometry to detect changes across small areas of interest.

The slices were mounted by the root of the tooth section onto a travelling platform to be scanned in two orthogonal directions. Regions of interest (Fig. 1) were selected visually according to where the artificial lesion was made. A Topcon telescope (AT-G2, Japan) was used to identify precise regions located using the telescope's cross hair which was aligned with the plane of the X-ray beam.

Each diffraction pattern took 20 s to collect (including 8 s CCD camera processing), and a total of 4839 diffraction patterns were collected.

#### 2.2.2 Data analysis

2D diffraction patterns of dentine and enamel can be distinguished by the Debye ring characteristics. Patterns averaging over 20.0 µm×20.0 µm, representative of dentine display uniformity in intensity azimuthally indicating lack of preferred orientation, and broadening of the Bragg peaks due to small crystallites. However, enamel diffraction patterns display variations in the intensity azimuthally especially around the (002) Bragg reflection which is indicative of the presence of preferred orientation [17], [31], [32].

The 2D diffraction patterns were pre-processed using the ESRF software package Fit2D (V12.077, France) [33]. Instrument parameters were determined using a LaB_6_ powder standard. Each diffraction pattern was azimuthally integrated over 360° in a narrow 2θ band containing the (002) reflection. Intensity versus the azimuthal angle was plotted and fitted to a Gaussian peak-shape. Changes in the full width half maximum (FWHM) in each azimuthal plot were monitored along a track (Fig. 1) from the enamel surface to the EDJ. Variations in the 2θ position of Bragg reflections provide information on changes in crystal chemistry as a result of different treatments [17]. The (002) Bragg reflection located at 2θ_(002)_ = 13.676° was used to measure changes in the percentage peak position.

### 2.3 Scanning Microradiography – Mineral Concentration

The SMR system has been developed over a number of years at Queen Mary University of London for direct and accurate mineral concentration measurements [26]–[30]. The X-ray source is a microfocussed X-ray generator (Ag target 40 kV, 1.0 mA tube current) with a small aperture to create an X-ray beam of ∼15 µm diameter [34]. SMR cells containing the tooth slice were constructed from a polymethyl methacrylate sheet and then mounted on the X-Y translational stage [28]. The number of photons in pre-set times at each scan position and outside the tooth slice were counted using a high purity geranium detector with a pulse height analyser (EG & G, Oak Ridge, TN, USA) set to pass AgKα radiation. High resolution area scans (stepsize 20 µm) were carried out on all slices. The projected mass of enamel at each position was calculated from the transmitted X-ray intensity, and published values of the mass attenuation coefficient of hydroxyapatite as previously described [26]. Although the absolute values of mineral concentration at each point were difficult to determine as this is dependent on specimen thickness, the tooth slices were planoparallel and therefore relative differences in mineral concentration arising from acidic attack at a particular point could be compared with unaffected points in the same slice.

### 2.4 Scanning Electron Microscopy - Microstructure

The microstructure of the same regions of interest were characterised using SEM. Each tooth slice was etched using 35% orthophosphoric acid for 15 s, (a mild and common procedure used in dental clinics to remove surface contaminants and the smear layer [35]), washed with distilled water then mounted on an aluminium stub and sputter-coated with gold. Microstructural images were collected using a field emission analytical SEM, Inspect F (FEI, Eindhoven, The Netherlands).

## Results

### 3.1 Synchrotron X-ray diffraction - Crystallography

#### 3.1.1 (002) Preferred Orientation

**Figure 2:**
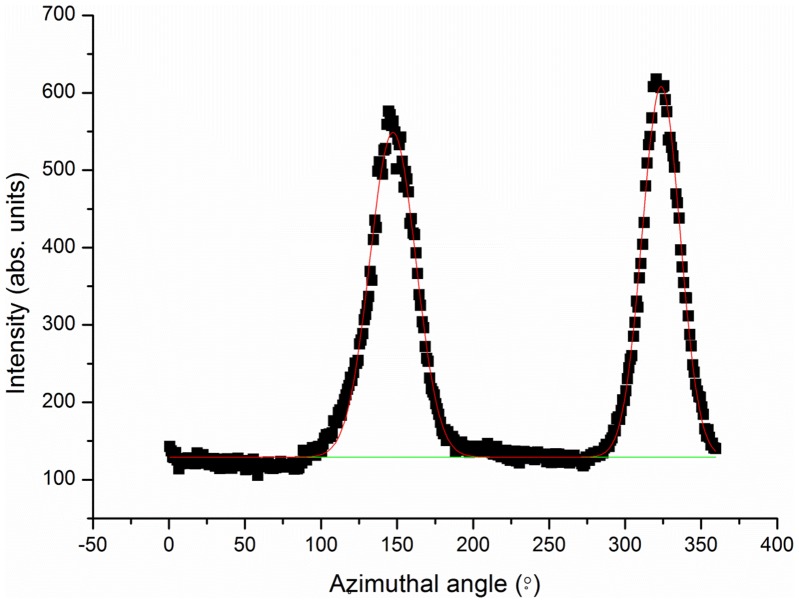
Intensity versus azimuthal angle taken from a typical (002) Bragg reflection of an enamel diffraction pattern. Pronounced peaks highlight a high degree of texture in this sample. Both peaks have been fitted to a Gaussian plus baseline (red and green lines respectively).

The (002) Miller index is orthogonal to the *c* axis of the hexagonal crystallite structure in hydroxyapatite. Since this is the long axis of the needle-like enamel crystallites, evidence of preferred orientation is most strongly seen as variations in the intensity around the Debye-ring of (002) Bragg reflection [32]. A typical plot of intensity versus azimuthal angle, fitted with a Gaussian peak shape to fit the FWHM, is shown in Fig. 2. Sharp intense peaks show the presence of strong preferred orientation (low values of FWHM), whereas broad peaks (high values of FWHM) would have indicated a more randomly distributed orientation of crystallites [36]. This analysis was applied to every diffraction image from each tooth slice across a track from the enamel surface to EDJ.

**Figure 3:**
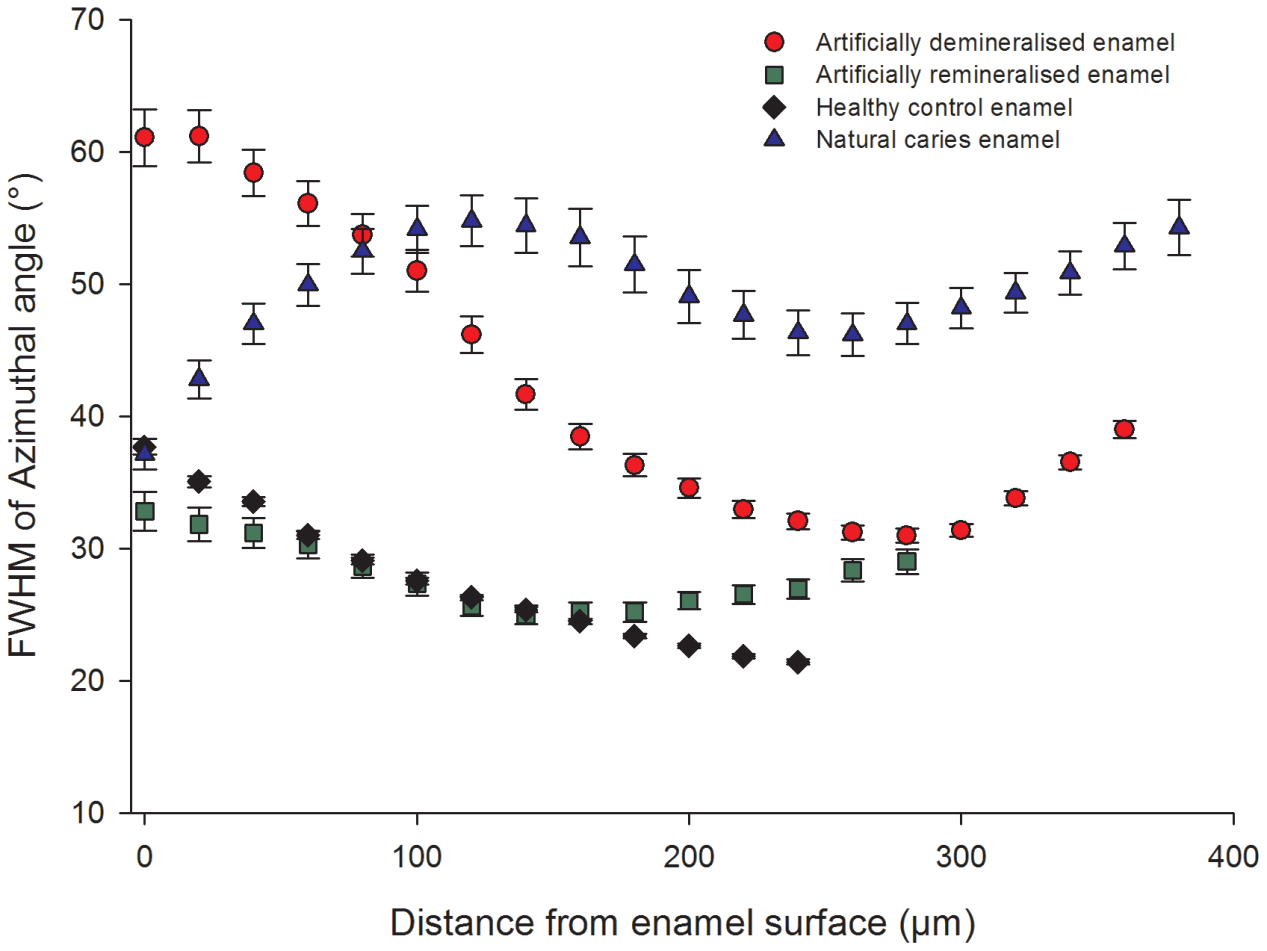
Changes in the FWHM. A track (highlighted in Fig. 1) taken through each tooth slice (artificial demineralization, artificial remineralization, healthy control and natural caries) from enamel surface to EDJ measuring the change in FWHM of the azimuthal peaks of the (002) reflection.

Fig. 3 shows the changes in the crystallographic texture (FWHM) of the (002) reflection (from the track shown in Fig. 1) as a function of position for all four tooth samples. Healthy enamel shows a gradual decrease in FWHM from the surface to deeper within the enamel. The first 80 µm from the surface of the artificially demineralized enamel exhibits significantly higher FWHM values, followed by a steep decrease moving further away from the surface until a depth of 275 µm, after which there is a slight rise. The first 20 µm of the natural caries enamel has similar FWHM values to that of healthy enamel with the highest values seen between 80–100 µm. In the artificially demineralized and subsequently remineralized specimen, the surface enamel has FWHM values similar to that of healthy enamel, and continues to follow the same trend as healthy enamel for most of the track, deviating slightly towards the EDJ.

FWHM data is plotted up to 400 µm from the surface, as this is the region which has been affected. Furthermore, the thickness of enamel varied according to the region that was studied. It must also be noted that the remineralized enamel was initially artificially demineralized which is a possible explanation for the increase in FWHM deeper into the enamel.

#### 3.1.2 (002) Bragg Peak position

**Figure 4:**
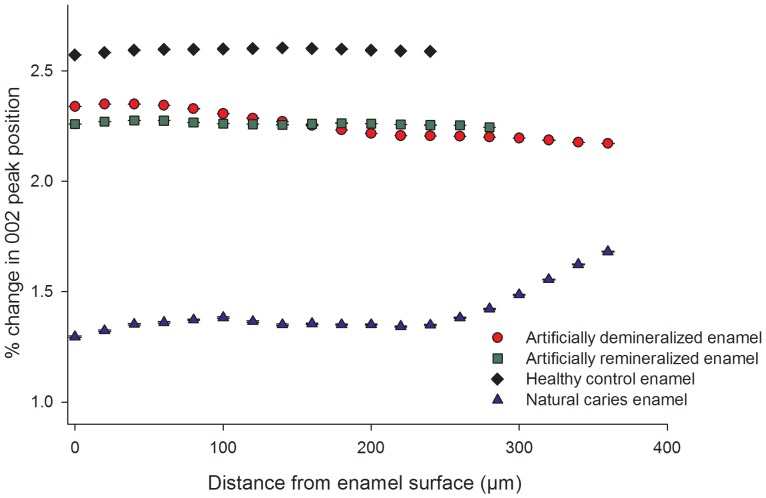
Changes in (002) Bragg peak position in a track (highlighted in **Fig. 1**) through each tooth slice (artificial demineralization, artificial remineralization, healthy control and natural caries) from enamel surface to EDJ.

Fig. 4 shows the percentage change in the (002) Bragg peak position as a function of distance from the natural tooth surface. There is very little change in peak position within each sample. A slight increase in the 2θ value is seen in the natural caries enamel from a 280 µm depth (<0.5%) onwards.

### 3.2 Scanning Microradiography - Mineral Concentration

**Figure 5:**
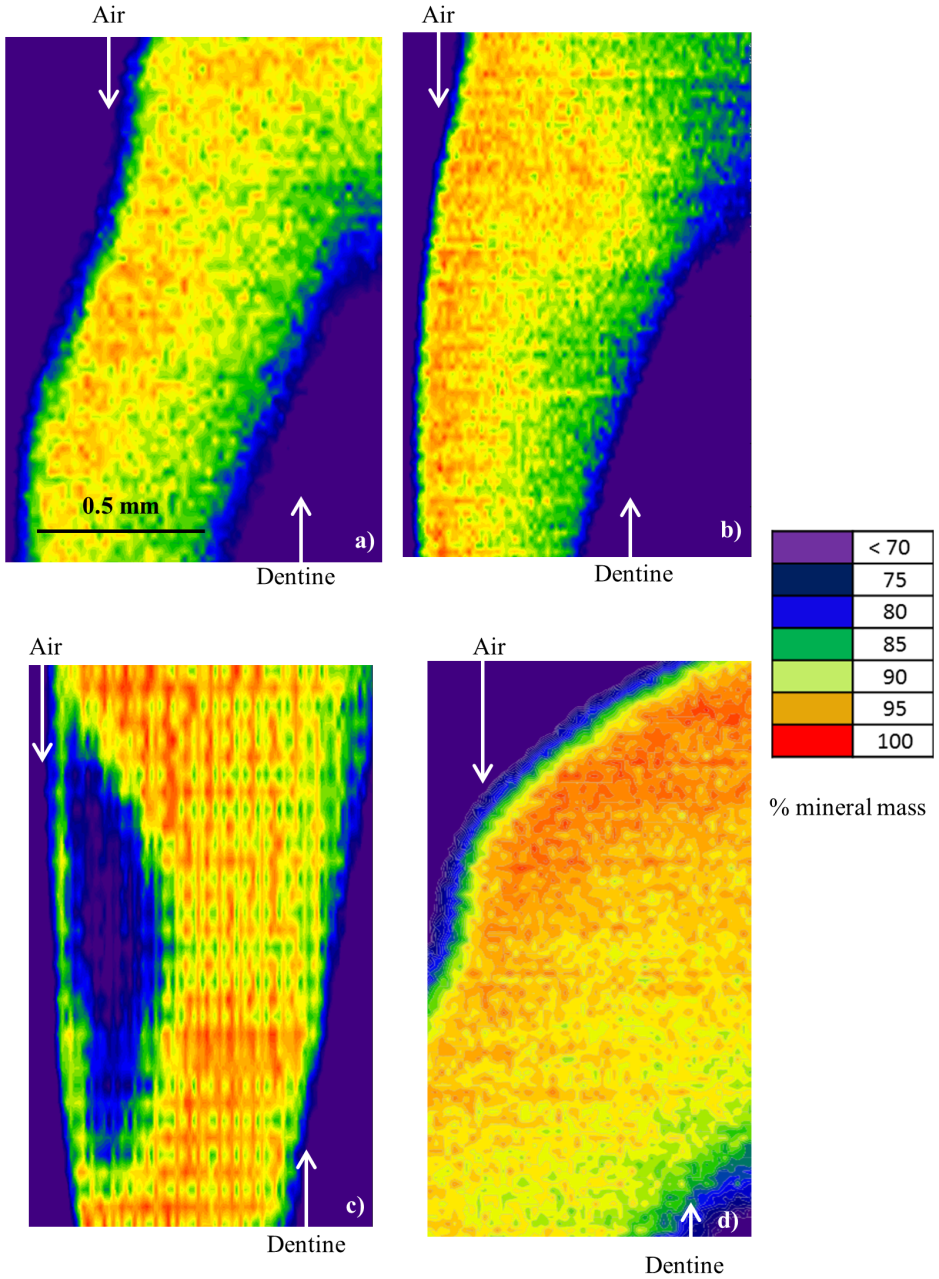
Scanning microradiography area scans showing the relative mineral density distribution of a) artificially demineralized lesion, b) artificially remineralized enamel, c) natural caries lesion, d) healthy enamel. The scale bar and colour intensity bar are representative for all images.

Fig. 5 shows the SMR area maps of the percentage change in mineral concentration for each slice. The maximum value for each enamel slice was defined as 100% mineral concentration in the unaffected region away from the lesion. The artificially demineralized enamel regions (Fig. 5a) can be differentiated from the healthy regions. For example Fig. 5c, shows a 10–30% lower mineral mass in the body of the lesion. Fig. 5b shows the artificially remineralized enamel and the uniformity of the mineral concentration across the tooth is such that the treated area is indistinguishable from the unaffected areas. The naturally carious (Fig. 5c) enamel shows a thin, low mineralized barrier isolating the subsurface lesion with an average 30% mineral mass reduction. Mineral density of the surrounding enamel is higher and uniformly distributed. The control, healthy enamel (Fig. 5d) shows uniform high mineral concentration. It should be noted that the thickness of the slices (500 µm) results in partial volume edge effects at the periphery, but this does not interfere with the measurement of the bulk enamel.

### 3.3 Scanning Electron Microscopy - Microstructure

**Figure 6:**
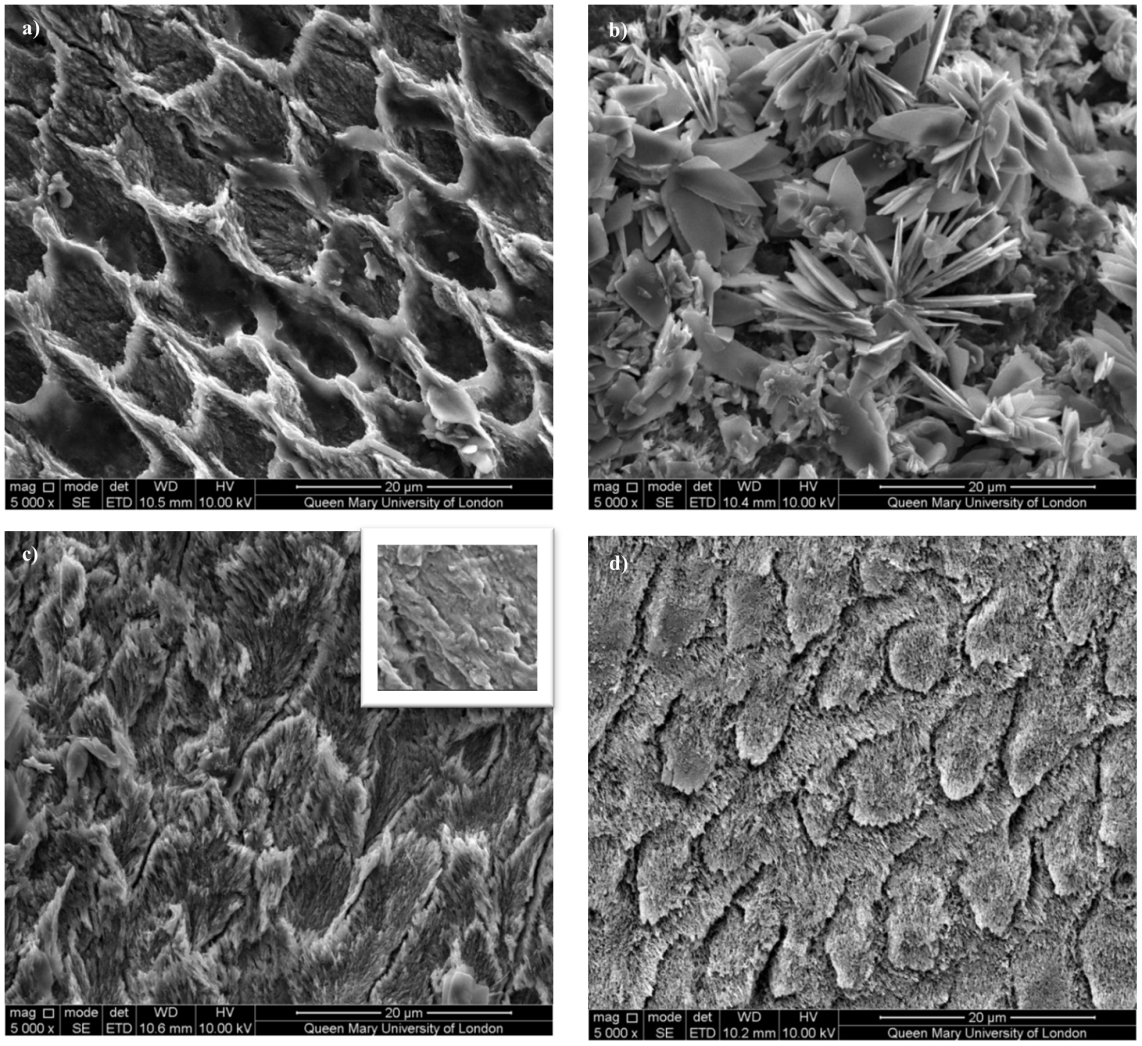
SEM images of enamel from the surface of a) artificially demineralized b) artificially remineralized c) naturally carious (surface) and d) healthy region. The inset to d) shows the bulk of the carious region ∼150 µm from the surface.

Fig. 6 shows representative SEM images close to the natural surface of the named regions in each specimen. The prisms in artificially demineralized enamel (Fig. 6a) appear etched where prism cores have been significantly more affected than the walls of the prisms and the interprismatic enamel which remain more intact. The image for the remineralized enamel (Fig. 6b) shows thin plate-like deposits arranged in clusters (lacking directional growth), forming a layer over the entire surface of the enamel specimen, with some small crystallites visible underneath. The crystallite morphology of the outer surface of the naturally carious enamel (Fig. 6c) resembles healthy enamel (long, thin, ordered crystals). However, moving in towards the bulk of the enamel towards the body of the lesion (inset), there is a loss of microstructural organisation and crystallites have fused together giving an amorphous appearance, with no distinct boundaries. Healthy enamel (Fig. 6d) shows long thin, needle like, bundles of crystals with preferred orientation evident.

## Discussion

### 4.1 Enamel Crystallite orientation

The azimuthal variation in intensity of the scattering signal around the (002) reflection (Fig. 3) was related to the arrangement of enamel crystallites in the prisms running from the EDJ to the surface [17].

This study demonstrates that a loss of crystallographic texture occurs as a function of demineralization in the natural caries and artificially induced lesions. However this loss was seen at different spatial regions between the two specimens, indicating a difference in the mechanism of crystallite dissolution. The surface of the artificially demineralized enamel suffered the greatest mineral loss and also the highest degree of loss in preferred orientation, which was seen within the first 100 µm from the surface (Fig. 3, filled circles). The crystallographic trend seen in the natural caries enamel (Fig. 3, filled triangles) correlated with the corresponding SMR data (Fig. 5) and resembles that observed by polarized light microscopy in previous studies of caries progression [37], [38] where the outermost enamel can remineralize within the oral environment. The natural caries enamel showed crystallite order at the surface where the highest degree of loss in crystallite order occurs between 80–180 µm deep into the bulk of enamel. The surface layer is a characteristic feature of naturally carious lesions, with an intact surface, an increase in texture suggests that crystallite order was regenerated naturally in the oral environment. Fig. 3 (filled diamond) reveals that texture was regained as a result of remineralization. The similarity in FWHM values and the depth-profiles between remineralized enamel and the healthy control enamel suggests that remineralization promotes re-formation of crystals in an ordered manner to restore their original orientation. Dissolution roughens the enamel producing kinks, steps and defects on the apatite crystallite surface which can serve as nucleation sites. The Ca^2+^ and PO_4_^3−^ ions react with these sites allowing the crystallite to grow on existing crystallite remnants rather than the *de novo* formation of new crystallites [11], [39], [40]. Growth of partially dissolved crystallites can occur by the surface condensation of free molecules in a supersaturated solution onto larger particles [41]. This SXRD study confirms that calcium and phosphate do not simply precipitate on the surface but in fact integrate into the apatite crystallite structure giving it preferred orientation. Voegel (1977) and Tohda et al. (1987) identified the initiation of dissolution at the core of each individual crystallite, which progressed rapidly along the (002) plane and later developed laterally [42], [43]. This suggests that in early carious lesions the *a* and *b* axes of each crystallite retain some structural framework, allowing the core of the crystallites to be rebuilt during remineralization. Results from a recent SAXS study have provided some of the first evidence to show a framework scaffold is preserved at a nano length scale to allow structure and direction for the reconstruction of the destructed crystallites during dissolution [22]. The results presented here concur with these findings, quantifying the precise order restored.

Little change was observed in the (002) Bragg peak position in the healthy, artificially demineralized, and artificially remineralized regions (Fig. 4). However in the carious enamel a slight change in peak position was observed which can be attributed to the cyclical nature of demineralization and remineralization. The dynamics of pH and chemistry in the oral environment may alter the crystal chemistry over time with inclusion and substitution of other ions from the oral environment into the enamel lattice structure, which may also affect the texture.

### 4.2 Enamel Mineral concentration

The SMR contour maps (Fig. 5) show a loss in percentage mineral mass in both artificially demineralized enamel (Fig. 5a) and naturally carious lesions (Fig. 5c). The greatest mineral loss in the natural caries samples was seen at the subsurface, isolated between healthy enamel and a mineralized surface layer. Features of the natural caries lesion seen here were most probably a consequence of successive periods of cyclic de- and remineralization over differing time periods when the oral environment favours one or the other process [44], [45]. Important interactions of the outermost enamel with Ca^2+^ and PO_4_^3−^ions present in saliva and exposure to fluoride in toothpaste promote surface remineralization [39], whilst mineral can be lost throughout the bulk of the enamel. A fully mineralised surface inhibits access of the ions in to the body of the lesion, preventing remineralization of deeper parts of the lesion, thus forming a subsurface carious lesion [22]. The mineral loss in the artificial lesion (induced by a demineralizing solution for 7 days) displays a different trend to that of the natural caries lesion. A slight decrease in mineral mass (∼10%) is observed at the surface, which is the direct site of attack. This is due to the extreme condition of continuous exposure to the demineralizing solution. Mineral concentration in the artificially remineralized enamel (Fig. 5b) is regained throughout the bulk of the enamel to match that of the healthy region of the specimen.

### 4.3 Enamel Microstructure

The microstructural effects of dissolution vary between natural caries and artificially demineralized enamel [6]. The morphology of both inter- and intra-prismatic enamel is different in natural caries and artificially demineralized enamel. This study shows a loss of texture from the artificially demineralized enamel as seen in the diffraction data (Fig. 3). This can be explained by the SEM images shown in Fig. 6b, where the demineralized enamel looks very similar to a straight-forward etched enamel surface. The SEM images show that in artificial demineralization, dissolution occurs predominantly in the prism cores which contain the more highly ordered crystallites. Since the ordered crystallites are preferentially lost, the remaining structure from the prism boundaries will have a lower texture on average. The naturally carious tissue has lost order in different parts of the prismatic structure, due to the cyclical process of alternating repeated demineralization and remineralization events according to the changing oral conditions. Fig. 6c shows that the microstructure of naturally carious enamel following dissolution results in a general loss of prismatic structure in a region ∼150 µm from the surface of the tooth. Lower crystallite packing allows easier diffusion of acids and protons into the tissue and mineral ions out [7]. The absence of distinct boundaries suggests dissolution has resulted in loss of structure and fusion of crystallites, diminishing preferred orientation.

Although crystallographically similar, the microstructure of remineralized enamel shown in the SEM images (Fig. 6c) appears to differ from healthy enamel. The large plate-like crystallites deposited seem to have formed a layer over the enamel crystallites. SEM images probe only the surface topography, potentially masking the more subtle regrowth of crystallites underneath the surface. This limitation is overcome by using SXRD, which allows the measurement of crystallite arrangements as a function of depth within the sample. Thus, early studies using SEM alone would not have been able to demonstrate that artificial demineralization differs from natural caries, nor understand the crystallite order following remineralization.

### 4.4 Comparing mineral concentration to nano- and micro-structure

The general trend seen in the SMR mineral density maps (Fig. 5) correspond to the texture distribution observed in Fig. 3; regions exhibiting lower mineral mass, such as the body of the natural caries lesion and surface of the artificially demineralised enamel, are the regions which lose crystallite order, whereas regions without reduction of mineral mass show higher crystallite order. The artificially remineralized region shows the same mineral mass as that of the control region and higher crystallite order.

## Conclusions

Enamel is susceptible to changes during the caries process which alter the mineral content and crystallographic organisation and microstructure. Characterising and understanding the crystallographic properties of carious enamel is important when developing restorative materials or preventative therapeutics. In this study, the mineral density and crystallographic preferred orientation of enamel have been quantitatively evaluated after demineralization and remineralization using specialized X-ray techniques. This study demonstrates that enamel which has lost mineral density and crystallographic texture via apatite dissolution can be restored to its original healthy state by a careful remineralization regime.
